# Researches in Medico-Legal Chemistry

**Published:** 1838-01

**Authors:** 


Art. II.—*Die Gerichtlich-chemischen Untersuchungen. Eine praktische Auleitung für Aerzte: entworfen vom* Dr. C. Gusserow, prakt. Arzte zu Berlin.—*Berlin*, 1836.

*Researches in Medico-legal Chemistry: designed as a Guide for Medical Practitioners.* By Dr. C. Gusserow.—*Berlin,* 1836. 8vo. pp. 76.

Dr. Gusserow's treatise is entirely confined to the subject of chemical toxicology; and we may truly say of it, that it is one of the best which has yet appeared to serve as a guide for the detection of poisons by chemical analysis. The processes for the discovery of individual poisons are preceded by a well-written introduction, in which clear and concise rules are given for determining whether any unknown substance, presented for examination, be really a poison; and, if so, to what class it belongs. The author follows throughout the rigorous laws of induction, and teaches the experimentalist to arrive, almost unconsciously, at an exact knowledge of the particular nature of the substance which he is engaged in examining. These rules of generalization, which must always be followed in practice, are usually omitted in works on toxicology; and we are therefore pleased to find this deficiency so well made up in the unpretending treatise before us.

Dr. Gusserow confines his remarks to the analysis of those poisons which are most commonly met with, and which in general call for medico-legal examination. The reagents which he employs and recommends are for the most part such as may be easily obtained by the experimentalist. The requisite cautions are given for their use; and, above all things, our author advises that their purity should be determined before they are employed in any analytical process. We may observe, that, throughout, the tests which he uses are perfectly adequate to the satisfactory detection of the particular poison; but here and there he introduces a few which are not only superfluous, but which may prove sometimes injurious in the hands of those who are not very expert in these matters. Thus, in the analysis of mineral poisons, the use of the hydrosulphuret of ammonia might have been omitted altogether. The sulphuretted hydrogen gas answers every purpose; it is easily made; it does not itself colour the solution to which it is added; and, what is of greater importance, the gas will not redissolve some precipitates which are readily taken up by the hydrosulphuret. The use of lime-water as a test for arsenious acid is entirely exploded in this country, because there are other reagents which afford better and more certain evidence. The subsequent reduction of arsenite of lime with boracic acid and charcoal, or with oxalate of lime, is a more complex process than the method commonly pursued in this country, by the employment of black flux. The use of the "hydrogen test" for the reduction of arsenic, under all its combinations, does not appear to have yet reached Germany.

The plans recommended for the detection of poisons in mixtures containing organic matter are clear and satisfactory. They may, we think, throughout be safely relied on, since they are evidently derived from an extensive practical acquaintance with the subject of toxicological analysis. An easy and simple method for detecting opium, or, in other words, its constituent principles, morphia and meconic acid, is yet a desideratum in toxicology. We find each experimentalist recommending a different plan; and some of these processes, from their tediousness, suffice to discourage many from attempting the experiment. These considerations have induced us to extract Gusserow's method for the detection of morphia and meconic acid in mixtures abounding in organic matter.

"*a.* The solid contents of the stomach, and other substances, must be sliced, bruised, and treated with successive quantities of distilled water. The different liquids are to be collected, and the solid residue pressed. Too large a quantity of water should not be employed in this preliminary proceeding.

"*b.* The solid residue, after compression, must be treated with an excess of diluted muriatic acid. Distilled water is then to be added, and the whole allowed to digest for from eight to sixteen hours. At the end of this time the liquid may be poured off, and the residue again well washed and pressed.

"*c.* The liquids obtained by *a* and *b* are now to be mixed, and evaporated to perfect dryness in a water bath. All organic solutions suspected to contain opium may be treated with diluted muriatic acid, and, after filtration, at once evaporated to dryness.

"*d.* The dry mass, *c*, must be digested in two successive portions of pure boiling alcohol; the solutions filtered, and then evaporated to the consistency of syrup. Whatever residue is left on the filter should be saved for the subsequent extraction of meconic acid.

"*e.* The alcoholic solution thus obtained is now to be treated with liquor ammoniæ in excess, and set aside. If opium were present, morphia would be slowly deposited in a confusedly crystalline form. The crystals may be obtained pure by redissolving the deposit in alcohol, and allowing this solution to evaporate spontaneously. The mother liquor may be made to yield an additional crop by evaporating it to dryness, and treating it with a small quantity of alcohol. If the original alcoholic solution, *e*, should not yield any deposit with ammonia, it should be evaporated to dryness, and digested in the same way in a very small quantity of pure alcohol." (P. 72.)

" For the extraction of *meconic* acid, the residue mentioned under *d* is to be digested in a dilute solution of ammonia, filtered, and the excess of alkali neutralized by acetic acid. Acetate of lead is now to be added until there is no further precipitation. The precipitate, which is the meconate of lead, is to be collected on a filter, well washed, and then boiled for some time with very dilute sulphuric acid. On filtering this liquid, the sulphate of lead remains on the filter, while the solution of meconic acid passes through." (P. 75.)

This process appears to us to be far better than any which has yet been published. We are aware that the method for separating meconic acid presents nothing particularly new; but the whole description seems to us so clear that we think there are few who would not, with a little care, be able to demonstrate by it the presence of opium in the most complex mixture in which that poison may present itself for examination.

We recommend this treatise strongly to those of our readers who are acquainted with the German language. So far as it extends, it is one of the best works which has yet appeared on the subject of which it treats.

